# The Long-Term Efficacy and Safety of Carotid Artery Stenting among the Elderly: A Single-Center Study in China

**DOI:** 10.1155/2018/4707104

**Published:** 2018-09-12

**Authors:** Lan Wen, Suxia Wang, Lei Liu, Lin Chen, Jia Geng, Lei Kuang, Gangzhen Qian, Junjie Su, Kangning Chen, Zhenhua Zhou

**Affiliations:** ^1^Department of Neurology, Southwest Hospital, Third Military Medical University, Chongqing 400038, China; ^2^Department of Pain, Southwest Hospital, Third Military Medical University, Chongqing 400038, China; ^3^Department of Neurology, First Affiliated Hospital of Chengdu Medical College, Chengdu, Sichuan 610000, China

## Abstract

Compared to carotid endarterectomy, carotid artery stenting (CAS) is reportedly associated with higher perioperative risks in elderly patients. To verify the long-term safety and efficacy of CAS with embolic protection in elderly patients, we retrospectively reviewed the medical records of patients with carotid stenosis treated between January 2003 and March 2010 at the Department of Neurology of a large university hospital in China. We included patients with symptomatic, moderate, or severe carotid stenosis of atherosclerotic etiology (other etiologies were excluded), with a disability score ≤ 3 on the modified Rankin Scale, and who received CAS instead of carotid endarterectomy. The clinical endpoints studied were stroke recurrence and all-cause death. The 84 patients included in this study (median follow-up, 8.08 years) were stratified according to age at surgery (<70 vs. ≥70 years), and no significant between-group difference was found regarding baseline characteristics. Of the 14 patients (16.67%) who experienced a defined clinical endpoint, 4 (7.14%) were aged <70 years and 10 (35.71%) were aged ≥70 years (*P* = 0.002). Overall mortality was 14.29% (12/84), with 3 (5.36%) and 9 (32.14%) deaths among patients aged <70 and ≥ 70 years, respectively (*P* = 0.002). Heart disease and cancer accounted for most deaths. The two groups did not differ regarding stroke recurrence, disability score, or rate of in-stent restenosis (blockage ≥ 50%), but patients aged ≥70 years had a higher risk of mortality (odds ratio, 8.3684; 95% confidence interval, 2.048–34.202; *P* = 0.003), and age was an independent risk factor for death (odds ratio, 20.054; 95% confidence interval, 3.094–129.987, *P* = 0.002). Among elderly patients in Southwest China, CAS can effectively prevent stroke recurrence without increasing the risk of stroke-related death, but the risk of all-cause death is higher, with age as an independent risk factor. Careful patient selection is of key importance in the treatment of symptomatic carotid artery stenosis.

## 1. Introduction

The gradual increase in the incidence of cerebrovascular disease reflects the aging trend in many populations. Cerebrovascular death has become one of the three major disease-related causes of death worldwide. Approximately 15% to 20% of ischemic strokes are caused by carotid artery stenosis [1]. There are three treatment options for carotid stenosis, especially for stenosis in the initial segment of the carotid; these options are drug therapy, carotid endarterectomy (CEA), and carotid artery stenting (CAS). It is currently believed that carotid revascularization via CAS or CEA can reduce the incidence of new stroke by 5%–12% [2]. Many comparative studies have evaluated the outcomes of CEA and CAS, including the following: the Stenting and Angioplasty with Protection in Patients at High Risk for Endarterectomy (SAPPHIRE) trial; the Endarterectomy Versus Angioplasty in patients with Symptomatic Severe Carotid Stenosis (EVA-3S) trial; the Stent-Protected Angioplasty versus Carotid Endarterectomy (SPACE) study; the International Carotid Stenting Study (ICSS); and the Carotid Revascularization Endarterectomy versus Stenting Trial (CREST) [3–6]. All these previous studies reported that patients treated with CAS had a higher risk of stroke but a lower rate of myocardial infarction and that the difference between the long-term outcomes of CAS and CEA was not statistically significant.

In China, CAS is widely used, and the safety and efficacy of revascularization therapy in the elderly population have become the focus of various studies. A meta-analysis [7] of the EVA-3S trial, SPACE study, and ICSS included an age-based subgroup investigation and revealed that the estimated 120-day risk of stroke or death among elderly patients (aged ≥70 years) was twice as high after CAS than after CEA. CREST [8] revealed that the age threshold for similarity of CAS and CEA outcomes over a follow-up of 4 years was 64 years regarding the risk of stroke, compared to 70 years regarding the risk of the primary endpoint (the composite of any stroke, MI, or death during a 30-day periprocedural period or ipsilateral stroke through follow-up of up to four years). It is currently accepted that the outcomes of CAS and CEA are closely related to age and that, among elderly patients, the risks associated with CAS are greater than those associated with CEA. Thus, in the present study, we aimed to assess the long-term efficacy and safety of CAS among the elderly. For this purpose, we retrospectively reviewed the medical records of patients treated for carotid stenosis in a single institution in Southwest China and analyzed stroke recurrence, incidence of all-cause death, disability, and rate of in-stent restenosis later than 120 days after CAS.

## 2. Methods

### 2.1. Patients and Study Design

All patients described in the manuscript provided informed consent for undergoing the procedures. The requirement for informed consent was waived on account of the retrospective nature of our study and the fact that no identifiable data are presented. Upon review of the medical records (including baseline and clinical characteristics), the candidates for this study were recruited from among the patients with symptomatic, moderate, or severe carotid stenosis with atherosclerotic etiology, treated between January 2003 and March 2010 at the Department of Neurology of Southwest Hospital, which is affiliated to the Third Military Medical University. The degree of stenosis was determined according to the standard applied in the North American Symptomatic Carotid Endarterectomy Trial (NASCET) [9]. The study included only patients with a disability score of ≤3 on the modified Rankin Scale (mRS) and who received CAS instead of CEA because of technical, surgery-related reasons (e.g., stenosis at anatomical sites inaccessible to CEA) or because of the patients' refusal to undergo CEA. Disability was evaluated on the mRS, with a score < 1 indicating no significant disability and a score of 6 indicating death. Patients with nonatherosclerotic causes of stenosis, such as arteritis or aortic dissection, were excluded.

The retrospective review of medical records collected data on epidemiologic variables (e.g., age and sex), classical risk factors for cerebrovascular disease (hypertension, diabetes mellitus, ischemic heart disease, alcohol consumption, and smoking), and risk factors for stroke recurrence (multiple arterial stenosis and plaque instability). Because the study aimed to verify the safety and efficacy of CAS among the elderly, the patients were stratified according to age at surgery (<70 vs. ≥70 years). The two groups were compared in terms of baseline characteristics and incidence of endpoints, and the potential risk factors for long-term mortality after CAS were evaluated.

### 2.2. Clinical Endpoints

The clinical endpoints analyzed in this study were stroke recurrence (any stroke), death, and the combined endpoint of any stroke or death later than 120 days after the surgery (until the end of follow-up). Stroke was defined as an acute deficit of focal neurological function with symptoms lasting for longer than 24 h, resulting from intracranial vascular disturbance (ischemia or hemorrhage). Visual loss that resulted from retinal ischemia and lasted for longer than 24 h was also considered a stroke endpoint. In-stent restenosis was defined as stenosis with blockage ≥ 50%.

### 2.3. Procedures

The patients were followed up by neurologists at the outpatient clinic to monitor for recurrent stroke (ipsilateral or contralateral) and assess the functional outcomes in terms of the mRS score. For patients who died during follow-up, relevant clinical information including the time and cause of death were recorded. Carotid ultrasonography was performed by sonographers in available patients. If restenosis was suspected, computed tomography angiography or digital subtraction angiography was performed for confirmation.

### 2.4. Statistical Analyses

All analyses were carried out using the SPSS statistical software package, version 22.0 (IBM Corp., Armonk, NY, USA). Age data had nonnormal distribution and were represented as median (interquartile difference). Frequency (%) was used to represent count data including age and stenosis severity distribution, as well as the incidence of hypertension, diabetes mellitus, alcohol consumption, ischemic heart disease, smoking, multiple stenoses, plaque instability, death, ischemic stroke, and mRS score < 2. Fisher's exact test and the chi-square test were used to evaluate the differences in baseline characteristics and clinical endpoints between the two groups defined in terms of age at surgery (<70 vs. ≥70 years). Univariable and multivariable logistic regression analyses were used to evaluate potential risk factors for death, including age group, sex, hypertension, diabetes mellitus, alcohol consumption, ischemic heart disease, smoking, multiple stenoses, stenosis severity, and plaque instability; the results were expressed as odds ratios (OR) with 95% confidence intervals (95% CIs). In all analyses, the level of significance was set at a *P* value of <0.05.

## 3. Results

### 3.1. Baseline Characteristics

Of the 98 patients that were considered candidates based on the inclusion and exclusion criteria, 84 had complete clinical and follow-up data and were thus included in the study. The median age in our study sample was 65 years (interquartile range, 20–26 years), 66 patients (78.57%) were male, and 28 were older than 70 years at the time of surgery. The distribution of carotid stenosis risk factors and associated diseases is summarized in Table 1. A total of 55 patients (65.48%) had hypertension, 15 (17.86%) had diabetes mellitus, 8 (9.52%) had coronary disease, 19 (22.62%) had multiple stenoses, and 62 (73.81%) had unstable plaque. There were no statistically significant differences regarding baseline characteristics between patients aged <70 years and those aged ≥70 years.

### 3.2. Clinical Endpoints

Over a median follow-up period of 8.08 years (interquartile range, 6.83–10.45 years; maximum, 14.1 years), there were no significant between-group differences regarding prevalence of mRS score < 2, stroke recurrence, or restenosis rate. However, death and the combined clinical endpoint of stroke or all-cause death had a higher incidence in the group of older patients (≥70 years). A total of 14 patients (16.67%) experienced the combined clinical endpoint (any stroke or all-cause death). Specifically, 4 of 56 patients (7.14%) aged <70 years and 10 of 28 patients (35.71%) aged ≥70 years had stroke recurrence or died during the defined period (*P* = 0.002). A total of 12 patients (14.29%) died, of whom 3 (5.36%) aged <70 years and 9 (32.14%) aged ≥70 years, indicating that advanced age is associated with increased mortality rate (*P* = 0.002). Stroke recurrence occurred in 3 of 84 patients (3.57%); specifically, 1 of 56 patients (1.79%) aged <70 years and 2 of 28 patients (7.14%) aged ≥70 years had stroke recurrence (*p* = 0.256), indicating that advanced age is not associated with increased rate of stroke recurrence. The prevalence of mRS score < 2 (not more than mild disability) was similar among patients aged <70 years and those aged ≥70 years (28.57% vs. 35.71%, *P* = 0.504). In-stent restenosis with blockage ≥ 50% occurred in 2 of 72 patients (2.78%) followed up at the outpatient clinic. Among patients aged <70 years at the time of surgery, 1 (1.18%) had in-stent restenosis with >70% blockage; among patients aged ≥70 years, 1 (5.26%) had in-stent restenosis with blockage of approximately 60% (Table 2).

### 3.3. Risk Factors for Death

Univariable logistic regression analysis indicated that, compared to patients aged <70 years, those aged ≥70 years were at higher risk of death (OR = 8.3684, 95% CI = 2.048–34.202, *P* = 0.003) (Table 3). To identify independent risk factors for mortality, multivariable logistic regression models were constructed using the following variables: age, sex, hypertension, diabetes mellitus, alcohol consumption, ischemic heart disease, smoking, multiple stenoses, stenosis severity, and plaque instability. The analysis revealed age to be the only independent risk factor for mortality (OR = 20.054, 95% CI = 3.094–129.987, *P* = 0.002).

### 3.4. Survival Duration and Causes of Death

**Figure 1:**
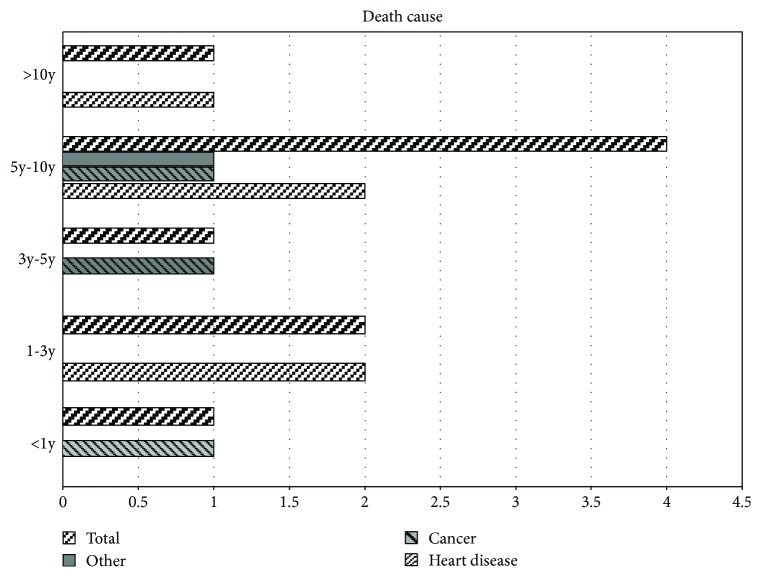
Analysis of survival and cause of death among elderly patients (≥70 years) who underwent carotid stenting. Deaths that occurred later than 120 days after surgery were stratified according to survival duration (<1 year, 1–3 years, 3–5 years, 5–10 years, and >10 years. Different columns represent different causes of death, with “other” indicating death not related to heart disease or cancer.

Twelve deaths occurred during the study period. Of the 9 elderly patients (aged ≥70 years) who died, one had survived for less than 1 year after CAS, while 6 patients had survived for more than 3 years and 1 had survived for more than 10 years. Among patients aged ≥70 years at the time of surgery, heart disease was the cause of death in 5 cases, cancer was the cause of death in 3 cases, and only 1 death had a different cause (neither heart disease nor cancer) (Figure 1).

## 4. Discussion

Carotid stenosis is one of the most important causes of ischemic stroke and represents an independent risk factor for ischemic cerebrovascular events [10]. Three major treatment strategies are currently available for carotid stenosis (i.e., drug therapy, CEA, and CAS), each with specific strengths and weaknesses. CAS has been widely used in the Chinese population, and most previous studies have confirmed its safety and effectiveness, although it should be noted that such studies have focused on comparative analyses of CAS and CEA outcomes. Several trials have reported a higher perioperative risk of stroke with CAS than with CEA [4–6]. In CREST [11], which is the largest controlled trial to evaluate CAS outcome, patients treated with CAS had a higher incidence of stroke in the perioperative period but a lower incidence of myocardial infarction compared to the incidence of such events among patients treated with CEA. On the other hand, analysis of long-term follow-up data (10 years) of the CREST participants [12] revealed no significant difference between CAS and CEA regarding the risk of such endpoints. In addition, CAS was reported to be relatively safe compared to CEA even in patients with concomitant severe coronary disease or atrial fibrillation [13–15]. Therefore, CAS and CEA may provide distinct advantages, and the choice between these surgeries should be made only after careful evaluation of the specific circumstances of each patient [16, 17].

Recent studies evaluating the long-term outcomes of CAS (median follow-up, 1.2–7.4 years) found some differences between CAS and CEA regarding the incidence of certain endpoints, but these differences were not significant. In the Carotid and Vertebral Artery Transluminal Angioplasty Study (CAVATAS) [18], SPACE trial [19], and EVA-3S trial [20], the incidence of the combined clinical endpoint of any stroke or perioperative death among CAS recipients was 29.7%, 9.5%, and 11.1%, respectively. In the SAPPHIRE trial [3], the incidence of myocardial infarction, stroke, or death after CAS was 32.0%. In CREST [11], the incidence of periprocedural (myocardial infarction, stroke, or death) or postprocedural endpoints (ipsilateral stroke) among CAS recipients was 9.0% in the 5-year survival group and 13.4% in the 10-year survival group. ICSS [21] reported a stroke recurrence rate of 6.4%, which is higher than the incidence of the combined clinical endpoint of stroke recurrence (any stroke) or all-cause death found in our present study; moreover, many patients included in our study had no significant disability after CAS, which was reflected in the high prevalence of mRS scores < 2 (Table 2).

A meta-analysis of 5 randomized controlled trials including 2716 patients and covering a median follow-up of 62 months concluded that there may be a relationship between in-stent restenosis and stroke recurrence after CAS, and the incidence of restenosis with blockage > 70% was 10% [22]. In our study, only 2 of the 72 patients followed up at the outpatient clinic had in-stent restenosis with blockage > 50%, and there was no significant difference between the two groups (<70 vs. ≥70 years at surgery) (Table 2). These results confirm the long-term safety and effectiveness of CAS.

Some data have indicated that elderly patients should be treated with CEA, which, compared to CAS, is associated with fewer risks in this patient population. A meta-analysis [7] of the EVA-3S trial, SPACE trial, and ICSS found that, in patients aged ≥70 years, the estimated 120-day risk of stroke or death was twice as high for CAS than for CEA (12% vs. 5.9%, OR = 2.04, 95% CI = 1.48–2.2, *P* = 0.053 for the interaction; *P* = 0.014 for the trend). Per-protocol analysis revealed estimates of 10% and 4%, respectively, for the 30-day rate of stroke or death among patients aged ≥70 years (OR = 2.1, 95% CI = 1.5–3.1; *P* = 0.078 for categorical interaction; *P* = 0.013 for trend interaction). The authors of the meta-analysis therefore suggested that, for the treatment of symptomatic carotid stenosis, CAS should be avoided in patients aged ≥70 years, while both CAS and CEA may be safe in patients aged <70 years. The 4-year follow-up data from CREST [8] revealed CAS and CEA provide similar outcomes regarding stroke recurrence in patients aged up to 64 years, with the risk of stroke and death increasing with every 10-year increment in age at surgery. We found significant differences in mortality between patients aged <70 years and those aged ≥70 years. Univariable logistic regression also suggested that elderly patients (aged ≥70 years) were at higher risk of death. Finally, multivariable logistic regression indicated that age was the only independent risk factor for mortality (Table 3). Our present findings are consistent with these previous data that, compared to patients aged <70 years, those aged ≥70 years have a higher mortality and higher incidence of the combined clinical endpoint of stroke recurrence (any stroke) or all-cause death. However, other than mortality, no other variables showed significant between-group differences in our study, including stroke recurrence rate, prevalence of mRS score < 2, and incidence of in-stent restenosis (Table 2).

Upon analyzing the data regarding cause of death (Figure 1), we found that heart disease accounted for 5 and cancer accounted for 3 of the 9 deaths that occurred among the elderly patients included in this study. The reason that heart disease was the most common cause of death among these patients is that atherosclerosis is a systemic disease, and patients with carotid stenosis are more likely to have ischemic heart disease and are more prone to cardiac complications. The reason that cancer was the second most common cause of death in these patients is that the incidence of cancer is higher in aging populations. None of the patients in this study died of stroke. With the exception of one death that occurred within 1 year after surgery, the remaining 11 patients who died during the study period had relatively long survival, with 6 patients surviving for more than 3 years and 1 surviving for more than 10 years. Several factors might explain the increased mortality rate in patients aged ≥70 years, such as the length of follow-up (median of 8.08 years starting from an age that was already close to the average life expectancy) and the prevalence of age-related complications. Our results indicate that, although elderly patients have higher mortality, advanced age did not significantly affect CAS outcomes in terms of preventing stroke recurrence and maintaining as much freedom from disability as possible.

The poor outcomes previously reported for transfemoral CAS in elderly patients may be due to the specific changes induced by atheromatosis in the aortic trunk and supra-aortic vessels. Transcervical CAS with flow reversal for cerebral protection avoids such unfavorable effects, and this strategy might provide higher long-term safety and effectiveness in elderly patients aged ≥70 years [23]. Therefore, improving personnel training and technique, use of embolic protection, and careful patient selection may help reduce mortality risk after CAS in the elderly.

The present study has some limitations. First, the studied population was recruited from a single institution. Second, the sample was small. Third, our study was restricted to CAS recipients. In addition, some patients admitted to our department during the study period (2003–2006) were not included in the study because they were lost to follow-up or had missing data regarding the follow-up evaluations.

## 5. Conclusion

Among elderly patients in Southwest China, CAS for moderate-to-severe carotid stenosis can effectively prevent stroke recurrence without increasing the risk of stroke-related death but is associated with increased all-cause mortality. Age is an independent risk factor for mortality after CAS. Among elderly patients, the main causes of death after CAS for carotid stenosis are heart disease and cancer. The treatment for patients with symptomatic carotid artery stenosis should be selected according to the individual circumstances of each case.

## Figures and Tables

**Table 1:** Baseline characteristics of patients undergoing carotid artery stenting.

Characteristic		Total	Age < 70 years (*n* = 56)	Age ≥ 70 years (*n* = 28)	*χ * ^2^†	*P* value
Sex	Male	66 (78.57%)	44 (78.57%)	22 (78.57%)	<0.001	>0.999
Hypertension		55 (65.48%)	36 (64.29%)	19 (67.86%)	0.105	0.746
Diabetes mellitus		15 (17.86%)	10 (17.86%)	5 (17.86%)	<0.001	>0.999
Alcohol consumption		24 (28.57%)	15 (26.79%)	9 (32.14%)	0.263	0.608
Ischemic heart disease		8 (9.52%)	4 (7.14%)	4 (14.29%)	/	0.431^∗^
Smoking		40 (47.62%)	30 (53.57%)	10 (35.71%)	2.386	0.122
Multiple stenoses		19 (22.62%)	12 (21.43%)	7 (25.00%)	0.136	0.712
Stenosis degree	Moderate	46 (54.76%)	33 (58.93%)	13 (46.43%)	1.177	0.278
	Severe	38 (45.24%)	23 (41.07%)	15 (53.57%)		
Plaque	Unstable	62 (73.81%)	42 (80.77%)	20 (74.07%)	0.472	0.492

Patients were stratified according to age at surgery (<70 vs. ≥70 years). The two groups did not differ regarding baseline characteristics (*P* > 0.05). Data are given as number of events (frequency). †: statistics according to the chi-square test; /: no *χ*^2^ value was found; ^∗^: *P* value for Fisher's exact test inadequate for the chi-square test.

**Table 2:** Clinical endpoints of carotid artery stenting.

Endpoint	Total	Age < 70 years (*n* = 56)	Age ≥ 70 years (*n* = 28)	*χ * ^2^†	*P* value
Stroke or death	14 (16.67%)	4 (7.14%)	10 (35.71%)	/	0.002^∗^
Death	12 (14.29%)	3 (5.36%)	9 (32.14%)	/	0.002^∗^
Stroke	3 (3.57%)	1 (1.79%)	2 (7.14%)	/	0.256^∗^
mRS score < 2	26 (30.95%)	16 (28.57%)	10 (35.71%)	0.446	0.504
Restenosis#	2 (2.78%)	1 (1.89%)	1 (5.26%)	/	0.460^∗^

Patients were stratified according to age at surgery (<70 vs. ≥70 years). Data are given as number of events (frequency). mRS: modified Rankin Scale; #: 12 cases were excluded from this subgroup analysis because death occurred prior to other endpoints (<70 years, *n* = 53; ≥70 years, *n* = 19); †: statistics according to the chi-square test; /: no *χ*^2^ value was found; ^∗^: *P* value for Fisher's exact test inadequate for the chi-square test.

**Table 3:** Results of the univariate and multivariate logistic regression analyses to identify independent risk factors for death after carotid artery stenting.

Risk factor		Total	Survival *n* (%)	Death *n* (%)	Univariate logistic regression			Multivariate logistic regression		
					OR (95% CI)	Wald *χ*^2^‡	*P* value	OR (95% CI)	Wald *χ*^2^§	*P*-value
Age, years	<70	56	53 (94.64)	3 (5.36)	Reference			Reference		
	≥70	28	19 (67.86)	9 (32.14)	8.368 (2.048–34.202)	8.748	0.003	20.054 (3.094–129.987)	9.887	0.002
Sex	Male	66	57 (86.36)	9 (13.64)	Reference			Reference		
	Female	18	15 (83.33)	3 (16.67)	1.267 (0.305–5.267)	0.106	0.745	0.398 (0.032–4.998)	0.510	0.475
Hypertension		55	48 (87.27)	7 (12.73)	0.700 (0.201–2.438)	0.314	0.575	1.263 (0.219–7.285)	0.068	0.794
Diabetes mellitus		15	13 (86.67)	2 (13.33)	0.908 (0.177–4.645)	0.014	0.907	2.573 (0.203–32.634)	0.532	0.466
Alcohol consumption		24	22 (91.67)	2 (8.33)	0.455 (0.092–2.249)	0.934	0.334	0.732 (0.072–7.448)	0.069	0.792
Ischemic heart disease		8	7 (87.50)	1 (12.50)	0.844 (0.094–7.547)	0.023	0.880	0.477 (0.027–8.282)	0.258	0.611
Smoking		40	37 (92.50)	3 (7.50)	0.315 (0.079–1.261)	2.664	0.103	0.524 (0.065–4.241)	0.366	0.545
Multiple stenosis		19	17 (89.47)	2 (10.53)	0.647 (0.129–3.246)	0.28	0.597	0.472 (0.059–3.773)	0.501	0.479
Stenosis degree	50~69%	46	40 (86.96)	6 (13.04)	Reference			Reference		
	70~99%	38	32 (84.21)	6 (15.79)	1.250 (0.368–4.248)	0.128	0.721	0.859 (0.131–5.647)	0.025	0.874
Plaque#	Unstable	62	53 (85.48)	9 (14.52)	1.274 (0.248–6.538)	0.084	0.772	1.950 (0.17–22.334)	0.288	0.591

Patients were stratified according to age at surgery (<70 vs. ≥70 years). Elderly patients (aged ≥70 years) had a higher risk of mortality (OR = 8.3684, 95% CI = 2.048–34.202, *P* = 0.003), and age was an independent risk factor (OR = 20.054, 95% CI = 3.094–129.987, *P* = 0.002). OR: odds ratio; 95% CI: 95% confidence interval; ‡: Wald *χ*^2^ for univariate logistic regression; §: Wald *χ*^2^ for multivariate logistic regression.
